# Effect of Vitamin D Supplementation on Faecal Microbiota: A Randomised Clinical Trial

**DOI:** 10.3390/nu11122888

**Published:** 2019-11-27

**Authors:** Negar Naderpoor, Aya Mousa, Luisa Fernanda Gomez Arango, Helen L. Barrett, Marloes Dekker Nitert, Barbora de Courten

**Affiliations:** 1 Monash Centre for Health Research and Implementation, School of Public Health and Preventive Medicine, Monash University, Clayton, VIC 3168 Australia; 2 Diabetes and Vascular Medicine Unit, Monash Health, Clayton, VIC 3168, Australia; 3 School of Chemistry and Molecular Biosciences, The University of Queensland, Brisbane, QLD 4101, Australia; 4 Mater Research Institute, The University of Queensland, South Brisbane, QLD 4101, Australia; 5 Department of Endocrinology, Mater Health, South Brisbane, QLD 4101, Australia

**Keywords:** randomised trial, 25-hydroxyvitamin D, faecal microbiota, inflammation

## Abstract

In animal studies, vitamin D supplementation has been shown to improve gut microbiota and intestinal inflammation. However, limited evidence exists on the effect of vitamin D supplementation on the human gut microbiota. We examined the effect of vitamin D supplementation on faecal microbiota in 26 vitamin D-deficient (25-hydroxyvitamin D (25(OH)D) ≤50 nmol/L), overweight or obese (BMI ≥25 kg/m^2^) otherwise healthy adults. Our study was ancillary to a community based double-blind randomised clinical trial, conducted between 2014 and 2016. The participants provided stool samples at baseline and after 100,000 international units (IU) loading dose of cholecalciferol followed by 4000 IU daily or matching placebo for 16 weeks. Faecal microbiota was analysed using 16S rRNA sequencing; V6–8 region. There was no significant difference in microbiome α-diversity between vitamin D and placebo groups at baseline and follow-up (all *p* > 0.05). In addition, no clustering was found based on vitamin D supplementation at follow-up (*p* = 0.3). However, there was a significant association between community composition and vitamin D supplementation at the genus level (*p* = 0.04). The vitamin D group had a higher abundance of genus *Lachnospira*, and lower abundance of genus *Blautia* (linear discriminate analysis >3.0). Moreover, individuals with 25(OH)D >75 nmol/L had a higher abundance of genus *Coprococcus* and lower abundance of genus *Ruminococcus* compared to those with 25(OH)D <50 nmol/L. Our findings suggest that vitamin D supplementation has some distinct effects on faecal microbiota. Future studies need to explore whether these effects would translate into improved clinical outcomes.

## 1. Introduction

Vitamin D deficiency is common worldwide mainly as a result of increased time spent indoors and increased use of sun protection to reduce the risk of skin cancer [1]. A growing body of evidence suggests extra-skeletal roles for vitamin D including insulin resistance, immune modulation, and inflammation [2]. The anti-inflammatory effects of vitamin D have been studied extensively in different conditions of acute and chronic subacute inflammation such as obesity, diabetes, and inflammatory bowel disease [3,4,5]. Faecal calprotectin, a marker of intestinal inflammation, has been shown to be inversely related to serum vitamin D concentration in Crohn’s disease [6]. However, findings from randomised clinical trials (RCTs) and observational studies on the effect of vitamin D on inflammation are inconsistent [3,5,7,8,9] and the underlying mechanisms are not fully understood. The effect of vitamin D on the gut microbiota has been proposed as a potential mechanism through which vitamin D may exert its role in insulin resistance and inflammation. The gut microbiota associations with chronic and inflammatory diseases have recently been recognised [10,11,12]. Limited evidence from animal and in vitro studies supports a bidirectional relationship between vitamin D and the gut microbiota [2,13,14,15]. Vitamin D has been shown to reduce bacterial infiltration into the colonic epithelium as well as bacterial-induced inflammation in animal models [2,16]. The absence of the vitamin D receptor (VDR) in VDR-knockout mice resulted in gut microbiota dysbiosis compared to the wild-type mice [17,18] and treatment with vitamin D in a different study ameliorated inflammatory lesions and symptoms in mouse models of colitis [15]. Vitamin D receptor stimulation in vitro and vitamin D supplementation in patients with Crohn’s disease has been shown to increase the secretion of antibacterial peptides such as cathelicidin and β-defensin 4A [5,19]. On the other hand, the gut microbiota has been reported to affect vitamin D metabolism and expression of the vitamin D receptor in the colonic epithelium [2,15]. However, very few observational and mechanistic studies have investigated the interactions between vitamin D and the gut microbiota and to our knowledge, there has been no previous RCT examining the effect of vitamin D supplementation on human faecal microbiota. 

We hypothesized that vitamin D supplementation alters the composition of the gut microbiota and may influence systemic and intestinal inflammation. Therefore, we aimed to compare the effects of vitamin D supplementation versus placebo on faecal microbiota, high sensitivity C-reactive protein (hs-CRP), and faecal calprotectin in vitamin D-deficient and overweight or obese individuals who are likely to be affected by subacute chronic inflammation.

## 2. Materials and Methods 

### 2.1. Study Design and Participants

This study was ancillary to a parallel-group, double-blind, randomised, placebo-controlled trial which was registered at clinicaltrials.gov as NCT02112721 [20]. The main outcomes of the study have been published [21]. In summary, we recruited volunteers from the community in Melbourne, Australia through advertisement. Inclusion criteria were as follows: aged 18 to 60 years, serum 25-hydroxyvitamin D (25(OH)D) concentration ≤50 nmol/L, body mass index (BMI) ≥25 kg/m^2^, and stable weight for the last 12 months prior to participation (<5 kg weight change) with no intention to lose weight. Exclusion criteria included any co-morbidities particularly diabetes, hypercalcaemia, and cancer within the preceding 5 years as well as taking any medications or supplements, smoking, alcohol intake >4 standard drinks (SD)/week for males and >2 SD/week for females, being pregnant, post-menopausal, or lactating, or the presence of acute inflammation based on history or blood test. Participants who were taking vitamin D supplements underwent a wash out period of three months prior to participating in the study. Ethics approval was granted by the Monash University Human Research Ethics Committee and Monash Health (ID: CF13/3874–2013001988). The study was conducted at a single centre and all participants provided written informed consent. 

At initial screening, participants underwent a physical examination including measurement of blood pressure, weight and height, and routine blood and oral glucose tolerance tests (75 g OGTT) to rule out any evidence of co-morbidities, diabetes, and acute inflammation. Two independent researchers randomised the participants using a computerized random-sequence-generation program in blocks of four by sex and season to receive a 100,000 international units (IU) loading dose of cholecalciferol orally followed by 4000 IU/day (four capsules) for 16 weeks or matching placebo. Participants were instructed to maintain their usual diet and exercise for the study duration. All participants as well as researchers who conducted the study and analysed the data were blinded until after the data were analysed.

### 2.2. Outcome Measures 

Body mass index (BMI) was calculated as weight (kg)/height squared (m^2^). Dual energy x-ray absorptiometry (DXA) (Lunar Radiation Corp., Madison, WI, USA) was used to assess body composition. Fasting venous blood samples were collected for 25(OH)D (direct competitive chemiluminescent immunoassays (DiaSorin Inc., Stillwater, MN, USA), inter- and intra-assay CVs of <10% and <4%, respectively), full blood counts (Beckman coulter LH750, Lane Cove, Australia), liver and kidney function tests (all using commercial enzymatic immunoassays, Beckman Coulter, Australia) and hs-CRP (sensitive near-infrared particle immunoassay rate methodology on a Synchron LX System Chemistry Analyser, Beckman Coulter, Australia). Faecal calprotectin was measured by sandwich immunoassay (Buhlmann, Switzerland). Dietary assessment at baseline and follow up was performed using the 3-day food diary and Foodworks 8.0 Professional; Xyris Software. Validated questionnaires were used to obtain data on physical activity (International Physical Activity Questionnaire [22]), and sun exposure [23] at baseline and follow up. Detailed description of the two questionnaires and calculation of physical activity and sun exposure index scores are published in our protocol [20]. Briefly, in the physical activity questionnaire, participants were asked to report the number of days, hours, and minutes engaged in vigorous and moderate activity, walking, and sitting during the previous seven days. Regarding the sun exposure, participants reported the average number of hours spent outdoors on a working and non-working day in summer and winter. In addition, they described clothing worn outdoors to help determine the fraction of body surface area (BSA) exposed to sunlight. A sun exposure index score was calculated as hours exposed to sunlight per week multiplied by the fraction of BSA exposed during that time [23]. 

### 2.3. Microbiome Profiling

Stool samples were collected at baseline and follow-up. Participants were instructed to keep the samples in the fridge if not delivered within four hours of collection. All samples were stored at –80 °C before the microbiota analysis. 

DNA extraction from 0.25 g of thawed stool sample was performed using repeated bead beating and column (RBB+C) method using sterile zirconia beads (0.1 and 0.5 mm diameter) with a 3 min mechanical disruption in 300 μL lysis buffer (NaCl 0.5 mol/L, Tris-HCL 50 mmol/L, pH 8.0, EDTA 50 mmol/L and SDS 4% w/v). Genomic DNA was further isolated using the Maxwell 16 Blood DNA purification kit following the manufacturer’s recommendations. A NanoDrop ND-1000 spectrophotometer (NanoDrop Technologies) was used to measure DNA concentration. The V6–V8 region was amplified and barcoded using the universal primers 926F (5’-TCG TCG GCA GCG TCA GAT GTG TAT AAG CAG AAA CTY AAA KGA ATT GRC GG – 3’) and 1392R (5’ GTC TCG TGG GCT CGG AGA TGT GTA TAA GAG ACA GAC GGG CGG TGW GTR C -3’) amplifying 500 bps of the 16S rRNA gene. PCR products were further purified, quantified, normalised, and pooled at The University of Queensland Australian Centre for Ecogenomics. The produced library was sequenced using Illumina MiSeq platform, according to the manufacturer’s instructions. 

Quantitative Insights into Microbial Ecology (QIIME) [24] version 1.9.1 software (www.qiime.org) was used to join, demultiplex, and quality filter the generated sequences. The operational taxonomic units (OTU) were picked using an open reference OTU picking method using 97% identity to the Greengenes 13_8 database. OTUs with a relative frequency below 0.01 were excluded. The resultant OTU table was normalised using the cumulative sum scaling (CSS) normalisation method. OTU tables at different taxonomic levels (phylum, class, order, family, and genus) were obtained. 

### 2.4. Statistical Analysis 

Statistical analyses were performed per protocol using SPSS (IBM version 24, Armonk, NY: IBM Corp) and the QIIME and Calypso software tools for microbiota analyses. Histograms and Shapiro–Wilk tests were used to assess whether variables were normally distributed. Data are reported as mean (standard deviation) for normally distributed variables or median (interquartile range) for variables with skewed distributions. Αlpha-diversity, defined as the total number of OTUs within one sample, was assessed using the paired Chao-1 index and Shannon index, which also evaluates the relative abundance of the various OTUs within the sample. Unsupervised ordination method (principal component analysis (PCA)) and supervised multivariate analysis (canonical correspondence analysis (CCA)) were used to identify significant differences in the microbiome composition between vitamin D and placebo groups (β-diversity). Differences in taxa at various levels were evaluated by Wilcoxon rank testing and linear discriminant analysis (LDA) effect size (LEfSe), where a higher LDA score reflected a more prominent difference in abundance between the vitamin D and placebo groups. The value of three was set as the significant differential threshold for the logarithmic LDA score [25].

The associations between the taxa abundance and anthropometric and inflammatory parameters were examined by bootstrapped Spearman rank correlation analysis. For all analyses, the Benjamini–Hochberg correction was performed in QIIME to adjust for false discovery rate (FDR) and to correct for multiple testing. 

In addition, a subgroup analysis was conducted to compare the faecal microbiome from individuals with vitamin D deficiency (25(OH)D <50 nmol/L) with those who achieved a 25(OH)D concentration higher than 75 nmol/L at follow-up as there is evidence suggesting that vitamin D supplementation has more beneficial effects at serum concentrations higher than 75 nmol/L [5,26]. 

For variables other than microbiota, differences between vitamin D and placebo groups were examined using independent Student’s *t*-tests or Mann–Whitney U tests (for non-normally distributed variables). A two-tailed *p*-value <0.05 was considered statistically significant. 

## 3. Results

**Figure 1:**
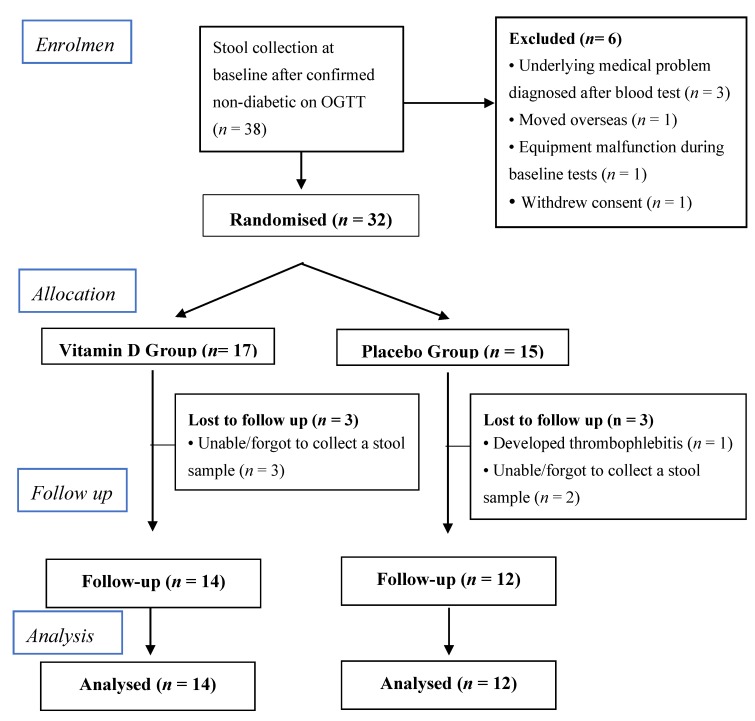
Consort flow chart for the microbiome study.

Figure 1 presents the CONSORT (Consolidated Standards of Reporting Trials) diagram of the participants’ flow. This sub-study included 38 individuals (22 males and 16 females), aged 18 to 57 years who provided stool samples at baseline. Six participants withdrew or had to be excluded prior to randomisation. Thirty-two participants were randomly assigned to receive vitamin D (*n* = 17) or placebo (*n* = 15) between September 2014 and July 2016. At follow-up, stool samples were received from 26 participants, 14 in the vitamin D and 12 in the placebo group (Figure 1). The participants’ characteristics at baseline and follow-up are presented in Table 1. There were no significant differences in baseline characteristics including serum 25(OH)D concentration, physical activity, sun exposure, total daily energy intake and dietary intake of carbohydrates, fat and proteins between the two groups. Baseline faecal calprotectin and hs-CRP were higher in the vitamin D group when compared to the placebo group. However, these were not statistically significant (Table 1). 

After 16 weeks of intervention, serum 25(OH)D concentrations were significantly higher in the vitamin D group when compared with the placebo (mean ± SD: 91.14 ± 25.8 vs. 31.58 ± 14.11 nmol/L) (Table 1). The vitamin D and placebo groups were not different in changes in BMI, % body fat, hs-CRP, or faecal calprotectin as well as in diet, physical activity, and sun exposure at follow-up (Table 1). There were no relationships between 25(OH)D and hs-CRP or faecal calprotectin at baseline or follow-up (*p* > 0.1 for all). 

### 3.1. Effect of Vitamin D Supplementation on Faecal Microbiota 

**Figure 2:**
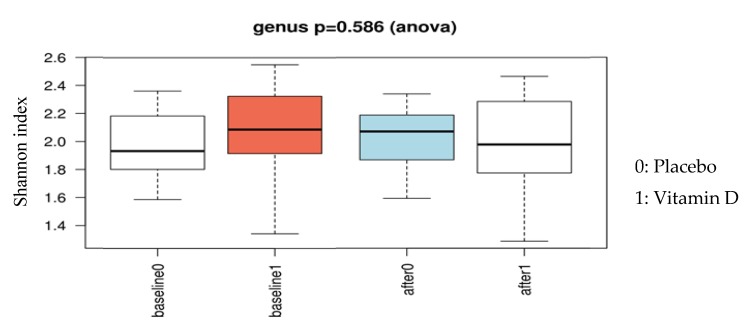
Comparing α-diversity at baseline and follow-up between the vitamin D and placebo groups.

**Figure 3:**
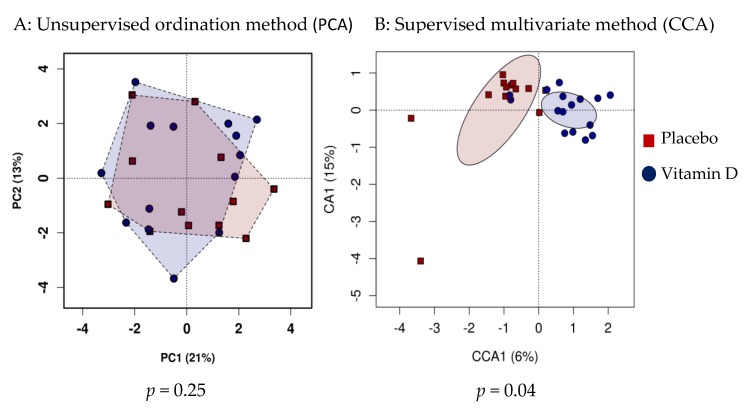
Comparing β-diversity at follow up between the vitamin D and placebo groups. PCA: principal component analysis, CCA: canonical correspondence analysis.

The alpha diversity of the microbiota profile at baseline was not different between the vitamin D and placebo groups (*p* = 0.9). Similarly, at follow-up, there were no significant differences in microbiota richness and evenness between the vitamin D and placebo groups (Chao 1 index *p* = 0.06, Shannon index *p* = 0.59, Figure 2). However, the vitamin D-supplemented group showed a reduction in bacterial richness at follow-up compared to the baseline (*p* = 0.050), whereas no significant differences were observed in the placebo group. Unsupervised hierarchical clustering analysis showed no significant clustering of the follow-up samples based on treatment allocation (*p* = 0.25, Figure 3A); however, there was a significant association between community composition and vitamin D 

**Figure 4:**
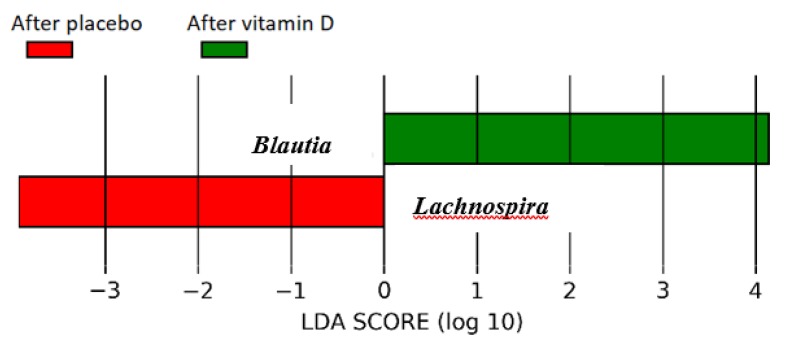
The Linear discriminant analysis Effect Size (LEfSe) plots demonstrating the significant differences in genera between the vitamin D and placebo groups at follow up and adjusted for multiple testing (the LDA (linear discriminant analysis) score threshold was set at three).

Regarding supplementation at the genus level in the supervised hierarchical clustering analysis (*p* = 0.04, Figure 3B), the vitamin D group had a significantly higher abundance of genus *Lachnospira* and lower abundance of genus *Blautia* compared to the placebo group after adjusting for multiple testing (linear discriminate analysis >3.0, Figure 4). There were no significant correlations between genus *Lachnospira* or *Blautia* and anthropometric measures (BMI, % body fat) or inflammatory markers (hs-CRP, faecal calprotectin) in the vitamin D or placebo group at follow-up (all *p* > 0.3). 

### 3.2. Subgroup Analysis 

**Figure 5:**
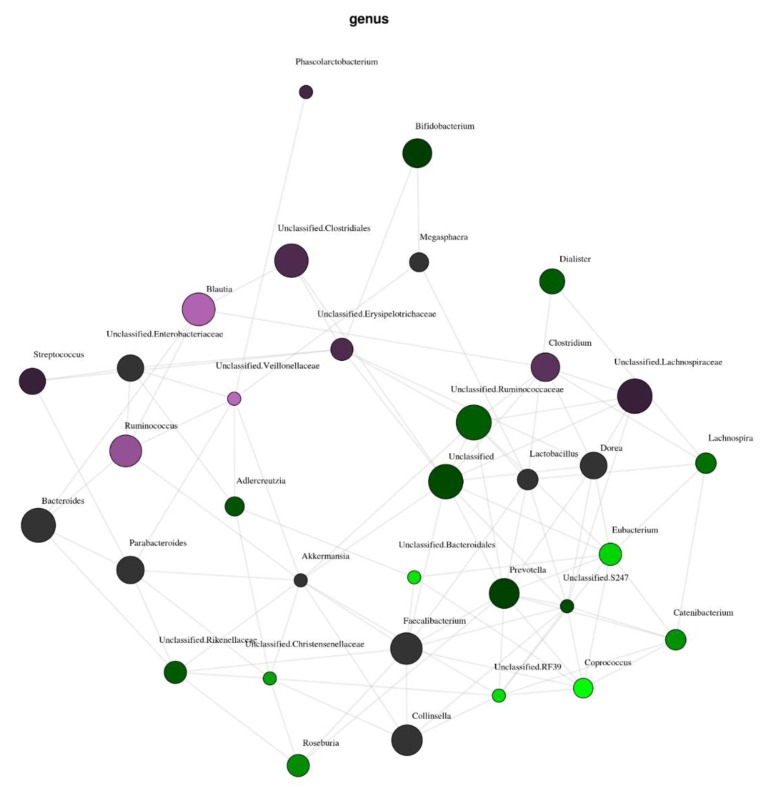
The network analysis illustrating the correlations of microbiota at the genus level with the low vitamin D (25(OH)D <50 nmol/L, *n* = 15, purple) and high vitamin D (25(OH)D >75 nmol/L, *n* = 10, green) groups at follow up. The node size indicates the overall abundance of the genus. The node colour intensity indicates the strength of the relationship (i.e., the brighter means a stronger correlation).

We compared the participants who had a serum 25(OH)D concentration lower than 50 nmol/L (*n* = 12) with those who achieved a 25(OH)D concentration higher than 75 nmol/L (*n* = 10) at follow-up. Only one participant from the vitamin D group had a 25(OH)D <50 nmol/L and none of the participants from the placebo group had a 25(OH)D >75 nmol/L. Figure 5 illustrates a network analysis of correlations of different genera with the two subgroups. There were no significant differences in α-diversity (individual samples diversity). Furthermore, the unsupervised analysis did not show any significant difference in β-diversity between the two groups. However, supervised analysis revealed a positive clustering of the samples based on 25(OH)D concentrations at the genus level (*p* = 0.04). Low vitamin D was associated with family *Clostridiaceae* (*р* = −0.54, *P* = 0.001), genus *Ruminococcus* (*р* = −0.51, *P* = 0.004). High vitamin D was associated with genus *Coprococcus* (*р* = 0.50, *P* = 0.01), and species *Coproccous eutactus* (*р* = 0.67, *P* = 0.02). We did not adjust for multiple testing due to the small population size. 

## 4. Discussion

We performed a randomised, placebo-controlled, double-blind study in a cohort of vitamin D-deficient overweight or obese otherwise healthy individuals and demonstrated the effects of vitamin D supplementation on faecal microbiota composition. We found that vitamin D supplementation was associated with higher abundance of genus *Lachnospira* and lower abundance of genus *Blautia*. Participants who achieved a 25(OH)D concentration above 75 nmol/L at follow-up had a higher abundance of genus *Coprococcus* and lower abundance of genus *Ruminococcus* compared to those with 25(OH)D concentrations lower than 50 nmol/L.

Serum 25(OH)D concentration has been shown in observational studies to be related to the abundance of specific bacterial genera. In a study of 3188 patients with inflammatory bowel disease, higher serum 25(OH)D concentrations were associated with lower risk of *Clostridium difficile* infection [27]. Another study by Luthold et al. involving 150 young healthy adults showed higher abundance of *Prevotella* and lower abundance of *Haemophilus* and *Veillonella* in those with highest tertile of vitamin D intake compared to others [9]. Vitamin D intake (through diet and supplements) was associated with serum 25(OH)D concentration in their study. They also reported inverse associations between 25(OH)D and *Coprococcus* and *Bifidobacterium*, which were attenuated after adjusting for inflammatory markers [9]. The effect of vitamin D supplementation on human gut microbiota has been examined in two previous open label pilot studies [28,29]. The first study examined the effect of vitamin D supplementation (5000 IU daily) for 90 days in seven females with multiple sclerosis (MS) and eight healthy controls. In agreement with our findings, faecal microbiota analysis at follow-up showed decreased abundance of genus *Ruminococcus* and additionally, increased abundance of *Akkermansia* and *Faecalibacterium* in this cohort [29]. In the subgroup analysis, MS patients who were not on glatiramer treatment showed an increase in abundance of *Coprococcus*, *Akkermansia,* and *Faecalibacterium* after vitamin D supplementation [29]. The second pilot study included 16 healthy volunteers (nine males and seven females) who received cholecalciferol for a total of eight weeks (140 IU/kg/day, maximum 68,600 IU/week for four weeks followed by 70 IU/kg/day, maximum 34,300 IU/week for the remainder of the study). The participants underwent endoscopies to examine biopsies from seven sites in the gastrointestinal (GI) tract and also provided stool samples for microbiota analysis [28]. This study reported a significant change in the gut microbiota in the upper GI tract (stomach and duodenum) with a decreased abundance of *Gammaproteobacteria* including *Pseudomonas* spp. and *Escherichia/Shigella* spp. as well as increased bacterial richness. However, no significant change was found in lower GI and faecal microbiome, which may be explained by the majority of vitamin D uptake occurring in the small intestine, particularly the jejunum and ileum [30]. Both studies had smaller samples sizes than our RCT and were not randomised or placebo-controlled.

Mechanistically, evidence from in vitro and in vivo studies also supports a role for vitamin D in gut microbiota composition and function as well as microbiota-induced inflammation and innate immune response [31]. All components of the vitamin D system including the VDR, vitamin D response elements (VDREs) and enzymes involved in the metabolism of active vitamin D (1,25 dihydroxyvitamin D_3_ (1,25(OH)_2_D_3_)) are present in colon epithelial cells [32,33,34]. VDR knock-out mice or mice that cannot produce 1,25(OH)_2_D_3_ have significantly different faecal microbiome composition compared to wild-type mice [18,35], suggesting a role for VDR and vitamin D in microbiota modification. Vitamin D_3_ supplementation to naked mole rats, which are naturally vitamin D-deficient, was shown to enhance microbial-controlled fermentation and production of short chain fatty acids in caecum [36]. In addition, vitamin D controls inflammatory responses to gut bacteria by modulating the antigenic signalling traffic between the gut microbiome and dendritic cells in the colon epithelium [18,37]. Cross-sectional studies of patients with inflammatory bowel disease have shown an inverse relationship between serum 25(OH) concentration and gut inflammation as measured by faecal calprotectin [6,38]. However, similar to our study, a pilot RCT involving 27 patients with Crohn’s disease in remission found no significant difference in faecal calprotectin after vitamin D supplementation compared to the placebo [5].

We found an increased abundance of *Lachnospira* and *Coprococcus* and a decreased abundance of *Blautia* and *Ruminococcus* with higher serum 25(OH)D concentrations. Some evidence from previous studies indicates a potentially beneficial effect for our observations. For instance, the abundance of *Lachnospira* has been reported to be lower in obese versus lean adults [39] and infants with lower abundance of *Lanchnospira* are at increased risk of developing asthma when compared to the controls [40]. These may suggest a beneficial effect of *Lachnospira* on BMI and immune response. Genus *Coprococcus* was shown to be increased in abundance in children living with pets [41] and in community-dwelling elderly when compared to aged care residents [42]. Additionally, its abundance is decreased in autistic children [43] and HIV patients [44]. Taken together, these findings indicate that *Coprococcus* abundance may be related to better health status. Furthermore, both *Blautia* and *Ruminococcus* genera have been reported to be associated with insulin resistance, higher HbA1c, and inflammation in other studies [45,46,47]. As such, our findings of reduced abundance of *Balutia* and *Ruminococus* after vitamin D supplementation in overweight or obese individuals may have a favourable impact on glycaemic control in this population. However, the significance of our results needs to be further assessed in future longitudinal studies.

In our RCT, vitamin D-induced alterations in faecal microbiota were not associated with any of the anthropometric outcomes or inflammatory markers. This is likely attributable to the small sample size and limited number of participants who achieved a serum 25(OH)D concentration above 75 nmol/L (*n* = 10).

To our knowledge, this is the first RCT to examine the effect of vitamin D supplementation on human faecal microbiota. The study had strict inclusion criteria that eliminated the confounding effects of co-morbidities, medications, alcohol, and smoking. Furthermore, the vitamin D and placebo groups were well-matched for sun exposure, physical activity, and diet composition at the baseline and there were no significant differences in these parameters at follow-up between the two groups.

However, the effect of vitamin D on faecal microbiota was a secondary outcome of this RCT and the sample size might not have been sufficiently powered to identify other potential differences between the vitamin D and placebo groups. Additionally, we only studied faecal microbiota, which may not fully represent the tissue microbiota or the microbiota residing in the upper GI tract. We did not analyse fresh samples and this may have impacted our results. However, all samples were stored in −80 °C within four hours of collection, otherwise were kept in the fridge and frozen in less than 24 h. Finally, our findings may not be generalizable to different populations such as lean individuals or those with an underlying inflammatory disease. To conclude, our study demonstrated a distinct impact of vitamin D supplementation on faecal microbiota in vitamin D-deficient overweight or obese adults, which may have favourable effects on BMI, insulin resistance, and inflammation in this group. This is the first RCT examining the effect of vitamin D on human faecal microbiota and our results as well as clinical significance of these outcomes need to be further demonstrated by additional RCTs. 

## Figures and Tables

**Table 1:** Baseline characteristics of the participants.

Variable	Vitamin D (*n* = 14)			Placebo (*n* = 12)			
	Baseline	Follow up	Change	Baseline	Follow up	Change	* *p*-Value
**Male/Female**	5/9			5/7			
**Age (years)**	34.36 (9.07)			32.75 (10.3)			
**BMI (Kg/m^2^)**	31.54 (4.4)	31.73 (4.1)	0.20 (0.5)	31.07 (4.1)	31.28 (5.3)	0.21 (1.4)	0.9
**% body fat**	40.26 (9.1)	37.4 (17.8)	−0.53 (1.4)	41.79 (10.0)	41.2 (18.2)	−0.37 (2.01)	0.8
**Daily energy intake (KJ)**	8076.61 (1910.1)	7641.19 (1769.5)	325.63 (2253.84)	8298.33 (4038.3)	7987.06 (2427.3)	−565.71 (2805.57)	0.5
**Daily carbohydrate intake (g)**	213.85 (54.9)	203.87 (77.4)	−18.88 (52.40)	230.20 (106.3)	193.80 (45.5)	−41.10 (64.58)	0.5
**Daily fat intake (g)**	74.53 (24.2)	68.99 (17.3)	0.47 (27.60)	73.93 (46.1)	77.88 (38.5)	−2.13 (30.86)	0.8
**Daily protein intake (g)**	92.31 (28.8)	84.52 (19.8)	1.10 (33.77)	60.54 (48.5)	99.32 (52.1)	1.15 (61.47)	0.9
**Sun exposure index score**	3.05 (2.2)	4.53 (2.8)	3.54 (6.12)	4.08 (3.4)	4.96 (6.3)	−2.18 (11.31)	0.1
**Daily physical activity (IPAQ MET score)**	2340.58 (1600.0)	1638.00 (1086.0)	−789.77 (1245.62)	3506.45 (2029.1)	3761.41 (3951.0)	−249.55 (4690.11)	0.7
**Faecal calprotectin (μg/g)**	12.00 (40.0)	6.50 (48.9)	0.00 (11.16)	5.95 (10.6]	11.50 (48.0)	4.10 (40.1)	0.5
**hs-CRP (mg/L)**	2.2 (3.6)	1.85 (4.8)	0.05 (1.00)	1.05 (3.1)	1.60 (2.3)	0.15 (1.30)	0.9
**25(OH)D (nmol/L)**	31.93 (12.7)	91.14 (25.8)	59.21 (26.67)	30.25 (11.2)	31.58 (14.11)	1.33 (8.50)	<0.001

Data are presented at mean (standard deviation) and median (interquartile range) for normally and not-normally distributed variables, respectively. BMI: body mass index, hs-CRP: high-sensitivity C-reactive protein, IPAQ MET Score: international physical activity questionnaire- multiples of the resting metabolic rate score. Sun exposure index score: Average sun exposure index for winter and summer calculated as hours sun exposure per week x fraction body surface area exposed. * *p*-value: for the differences in change scores at follow-up between vitamin D and placebo groups.
